# Increasing access to HIV testing for men who have sex with men in Japan using digital vending machine technology

**DOI:** 10.1177/09564624221094965

**Published:** 2022-05-03

**Authors:** Noriyo Kaneko, Nigel Sherriff, Michiko Takaku, Jaime H Vera, Carlos Peralta, Kohta Iwahashi, Toshihiko Ishida, Massimo Mirandola

**Affiliations:** 1 Department of Global and Community Health Nursing, School of Nursing, 12963 Nagoya City University, Nagoya, Japan; 2 School of Sport and Health Sciences, 1947 University of Brighton, Brighton, UK; 3 Centre for Transforming Sexuality and Gender, 1947 University of Brighton, Brighton, UK; 4 School of Nursing, 157621 Gifu University of Health Sciences, Gifu, Japan; 5 Centre for Global Health and Infection, 1949 Brighton & Sussex Medical School, University of Sussex, Brighton, UK; 6 Department of Art and Design, 7314 Sheffield Hallam University, Sheffield, UK; 7 akta, Tokyo, Japan; 8 ANGEL LIFE NAGOYA, Nagoya, Japan; 9 Infectious Diseases Section, Department of Diagnostics and Public Health, 19051 University of Verona, Verona, Italy

**Keywords:** HIV testing, self-sampling, Japan, vending machine, men who have sex with men

## Abstract

**Background:** In Japan, most new HIV cases are reported amongst men who have sex with men (MSM); thus, there is an urgent need for further widespread testing of MSM. The use of Digital Vending Machines (DVM) in the UK offering HIV test kits targeting MSM show promising results. Digital Vending Machines could be useful to promote and increase the uptake of testing in Japan, although no studies have yet been conducted. We aimed to assess the acceptability and feasibility of distributing HIV test kits using DVMs exploring needs and concerns as well as preferred types of test kits and locations.

**Methods:** Fifty-four individuals participated in workshops and meetings with a further 224 MSM answering a quantitative survey assessing HIV testing and prevention needs.

**Results:** Amongst MSM who had never been tested, 73% showed willingness to purchase tests from DVMs. Responses were broadly positive about DVMs but there were concerns regarding being seen receiving test kits from the machines and linkage to confirmatory testing and appropriate care.

**Conclusions:** Using DVMs to distribute HIV test kits in Japan was found to be both acceptable and feasible and may have the potential to increase access to testing for MSM. Future large-scale evaluation studies are required.

## Introduction

In 2019, 26.9% of newly diagnosed human immunodeficiency virus (HIV) cases in Japan were reported as acquired immunodeficiency syndrome (AIDS), representing a much higher rate than reported in Western developed countries.^1^ The 95-95-95 strategy aims to end the AIDS epidemic by 2030.^2^ To meet the first target, people with HIV must be tested and be aware of their status. However, this goal is yet to be achieved in Japan.^3^ The lifetime HIV testing experience in the key population of men who have sex with men (MSM) is 60–70%, which is much lower than in many Western countries.^4,5^ Barriers to the uptake and frequency of HIV testing in this population are relatively well known in Japan and other countries; for example, older age, perceived stigma, confidentiality concerns, delays in getting appointments, long waiting times in clinics, and having had no anal intercourse.^4,6,7^ Despite attempts to overcome these barriers in Japan,^8^ it is evident that the increased promotion of, and access to, HIV testing is both urgent and essential to meet the 95-95-95 target.

Providing opportunities for MSM to test using dried blood sampling (DBS) kits has been shown to improve the uptake of HIV testing.^9^ Many developed countries have now adopted such self-sampling and indeed self-testing methods.^10–12^ In Japan, DBS-based HIV testing kits are sold online by private companies. Although transactions are continuing, these testing kits are not yet officially sanctioned by the Japanese Ministry of Health, Labour, and Welfare as an approved HIV testing method. A DBS-based HIV testing kit distribution research project using the postal service was implemented by Akta which runs gay community centres ^13^ in Tokyo, in 2015–2017 and again in 2018-2020.^14^ Akta administered > 2000 test kits during the latter 2-year study period. The trials revealed that HIV prevalence exceeded 3%, which is 5–10 times higher than that noted in standard clinical facility-based testing or public health centres. Such findings strongly support the need for increased community-based HIV prevention and testing opportunities for MSM.^15^

In Japan, where the MSM population is relatively large, but the rate of disclosure of sexual orientation is remarkably low,^8,16^ effective and efficient ways to inconspicuously distribute HIV testing kits are required. In Brighton, United Kingdom (UK), digital vending machine (DVM) technology has been developed and implemented to increase access to HIV self-testing for MSM via gay saunas or bath houses.^17,18^ DVMs are abundantly available in Japan^19^; condoms are already sold in such machines^20^ along with cigarettes and alcohol on the streets of both commercial districts and residential areas. Therefore, DVMs could be particularly useful for widening the access to community HIV testing in Japan. Previously, the use of DVMs to promote HIV testing has shown some promising results across diverse populations worldwide.^17,18,21–24^ However, no studies have yet been conducted on the acceptability and feasibility of using DVMs to disseminate HIV self-testing kits in Japan. This could be particularly beneficial given the shortage of human resources dedicated to HIV prevention amongst the Japanese MSM population.^8^ Therefore, we aimed to assess the acceptability and feasibility of DVM-based test kit distribution qualitatively, exploring the end-user’s associated needs and concerns as well as advantages and disadvantages of the types of test kits that could be made available in this manner. We also aimed to quantitatively explore the acceptability or feasibility of using DVMs to distribute HIV test kits to MSM via commercial gay businesses.

## Materials and methods

A mixed methods study design was adopted to explore the perceptions regarding both acceptability and feasibility of using DVMs to distribute HIV self-testing kits (including self-sampling) amongst MSM in three Japanese cities (Nagoya, Osaka, and Tokyo). First, a participatory qualitative study was conducted involving MSM participants, gay non-governmental organisation (NGO) representatives, and commercial gay business (sauna) representatives. Second, a quantitative survey was performed to investigate the acceptability/feasibility of using DVMs to distribute HIV sampling kits to MSM via gay businesses.

### Qualitative workshop design

For the qualitative component of the study, 54 participants were engaged in three workshop discussions (*n* = 38) in 2019 (Table 1). To take part, participants had to be ≥ 20 years old, and identify as one or more of the following: a man who has sex with other men; a Community Health Worker (CHW) engaged in HIV prevention for MSM; an owner/manager of a commercial gay business; a researcher/academic focused on HIV prevention programmes targeting MSM. Additional discussions (*n* = 16) were held between Japanese and UK universities in collaboration with a local NGO based in Nagoya.

**Table 1.** Workshop characteristics.

	Location	Participating organisations	Country/prefectures represented	*n*
Workshop 1	Tokyo (*akta* community centre)	*akta*, gay business owner, Mr. Gay Japan, Nagoya City University (NCU), University of Brighton (UoB)	Japan: Tokyo, Kanagawa, Tohoku, United Kingdom (UK): Brighton	15
Workshop 2	Osaka (community space dista)	MASH Osaka, NCU and UoB	Japan: Osaka, Kyoto, UK: Brighton	11
Workshop 3	Nagoya (rise community centre)	Angel Life Nagoya, NCU and UoB	Japan: Aichi, UK: Brighton	12
			Total	**38**
Additional discussions and meetings				
Meeting 1 (NCU)	Nagoya	NCU and UoB	Japan: Aichi, UK: Brighton	5
Meeting 2 (NCU)	Nagoya	NCU, UoB, and Angel Life Nagoya	Japan: Aichi, UK: Brighton	6
Meeting 3 (NCU)	Nagoya	NCU and UoB	Japan: Aichi, UK: Brighton	5
			Total	**16**

The workshop discussions had two aims: first, to introduce participants to the concept of potentially distributing HIV self-test kits via DVMs based on the existing model used in Brighton, and second; to assess the feasibility of using such a system in the context of Japanese HIV care and the overall healthcare system. We also examined end-users concerns, barriers to implementation, facilitating factors, opportunities (e.g., increasing access to testing), and practical considerations associated with the types of test kits that could be made available (e.g. self-sampling vs self-testing, HIV and syphilis single tests vs HIV/Syphilis duo tests, and tests for other sexually transmitted infections (STI) such as chlamydia). The second aim of the workshop was to establish any design preferences and priorities for potential end-users, including the physical appearance of the machine, its digital interface, and potential locations.

To achieve these aims, each workshop divided participants into two groups. Each group was hosted by a Japanese University representative (MT, NK) who provided (where possible) simultaneous Japanese/English translation. Each workshop started with a presentation of the DVM and its current interface, delivered by the workshop facilitator (CP). In addition, the participants were instructed on how to download the machine interface mock-up on their mobile phones to help them experience what it would feel like to use the machine. Moreover, visual props were presented to the groups to elicit their opinions and preferences regarding the machine’s appearance, interface, and location.

### Quantitative survey design

For the quantitative component, in May 2019, a short survey was conducted at the Nagoya Lesbian & Gay Revolution + 2019 event, held in Nagoya, Japan, to determine the acceptability of DVMs amongst MSM attendees. This utilised self-administered paper-based questionnaire survey. Participation incentives included raffle participation with prizes, including ‘QUO’ cards (pre-paid gift cards that people had a 5% chance of winning, worth 8.0 US dollars as of 14/12/2021) and a choice of soft drink (1 US dollar). The survey included 26 questions organised into four categories as follows: (1) background; (2) HIV testing experience; (3) sexual behaviour; and (4) recognition of, and desire to, use DVMs. Category 4 included questions on the desire to buy an HIV testing kit from a DVM at a cost of 1000 Japanese yen (which has been determined to be an acceptable co-payment for HIV tests for MSM in Japan; see Kaneko & Shiono, 2020), and DVM location preferences.

### Ethical considerations

Workshops were audio-recorded with consent from all participants. Survey items were co-produced in collaboration with community centre staff. The research protocol was approved by the Institutional Review Board in the School of Nursing, Nagoya City University (15002-3).

### Data analysis

All conversations were transcribed and translated into English by the authors (NK and MT). The transcripts were analysed thematically using the framework approach.^25^ To enhance the credibility of the analytical process, the data were also analysed by the sixth and seventh authors (KI and TI), and all themes were discussed with the other authors to achieve consensus. The findings were sent to one participating group (akta) and the members subsequently provided feedback which was incorporated into the final analysis. Thus, our qualitative results represent a summary of findings from the series of workshop discussions, including a description of the major themes that emerged based on a detailed analysis of the transcripts.

### Quantitative survey

261 (87%) out of 300 distributed questionnaires were completed. Duplicate and incomplete responses were excluded. Subsequently, data from 222 gay and bisexual men were used for this aspect of the study. Participants were divided into two groups based on whether they had ever undergone an HIV test. Data tabulation and statistical tests were performed using IBM SPSS Statistics ver.22. The χ2 and Fisher’s exact tests were used to compare the responses between the groups; *p*-values < .05 were considered statistically significant.

## Results

### Perceived benefits and capability of using DVMs to distribute HIV testing kits

All participants of the workshops stressed the importance of the design and function of the DVM, using words like “cute”, “stylish”, and “Apple-like design” to describe its most appealing attributes. Promotion of the DVM could rely on “word-of-mouth”, which has a strong impact in the Japanese MSM community since most HIV testing information in Japanese society is based on the premise of heterosexual sex; gay and bisexual men thus gather and trust information from their peers. Therefore, participants stressed that it was highly effective to show social media messages from peers on a DVM interface. Another appealing attribute was that through DVMs, many testing kits could be distributed to a large number of people without involving staff. For clients who were not knowledgeable about testing and needed explanations and counselling, in-person distribution of test kits may be preferable; however, for those familiar with HIV testing, DVM-based distribution may be advantageous.

The participant characteristics and needs regarding self-testing DBS HIV kits stratified by HIV testing experience are shown in Table 2. 83.5% of participants had experience with HIV testing. Those who had been tested were more likely to be aware of DBS HIV kits and were more inclined to use them. Amongst those who had never been tested, 72.2% of participants expressed the desire to buy an HIV testing kit from a DVM in a gay sauna or bath house, even at a price of 1000 yen.

**Table 2.** Participants’ background and needs for using or buying self-testing/dried blood sampling HIV tests, stratified by testing experience (*N* = 224).

	Lifetime HIV testing experience				
	Never tested *N*^a^ = 37(16.5%)		Ever tested *N*^a^ = 187(83.5%)		*p*
	*n*	%	*n*	%	
Sexuality					
Gay	27	73.0	160	85.6	.060
Bisexual/not decided	10	27.0	27	14.4	
Age (mean ± SD)		28.9 ± 9.7		33.5 ± 8.9	-
Gay venue use^b^					
Yes	26	70.3	153	81.8	.110
No	11	29.7	34	18.2	
Condom use during last anal intercourse^c^					
Yes	7	22.6	78	43.3	.031
No	24	77.4	102	56.7	
Do you know of ST/postal DBS HIV testing?					
Yes	10	29.7	117	63.7	<.001
No	26	70.3	65	36.3	
Would you use ST/postal DBS HIV testing, if it were free?					
Very much	12	33.3	89	49.2	.017
Pretty much	16	44.4	51	28.2	
Not so much	8	22.2	22	12.2	
Do not want to	0	0	19	10.5	
Would you use DBS HIV testing/ST, if it cost 1000 Japanese yen?					
Very much	3	8.3	47	26.1	.007
Pretty much	19	52.8	67	37.2	
Not so much	13	36.1	39	21.7	
Do not want to	1	2.8	27	15.0	
Would you buy a 1000 yen worth of DBS HIV testing/ST kit provided by a DVM at the sauna?					
Yes	26	72.2	116	64.1	.350
No	10	27.8	65	35.9	

^a^Total numbers differ due to missing data.

^b^Includes gay bar, club event, shop, and sauna.

^c^Only amongst those who had anal sex in the past months.

DBS (dried blood sampling); DVM (digital vending machine); HIV (human immunodeficiency virus); SD (standard deviation); ST (self-testing).

### Points of concern

The most prominent concern raised in all workshops was about being seen taking a test kit from a DVM. “What will others think of me if they see me receiving a test kit from a DVM”? (Workshop 1) stirred strong opinions and agreement from several participants.

In addition, there were concerns regarding the receipt of a positive test result whilst alone. One NGO representative stated that “*In the UK, gay-friendly HIV and STI-related testing and treatment are widely available and provided free of charge, whereas in Japan the service provisions are limited. I am really worried about users with positive results not necessarily having immediate access to a confirmation test” Gay man in his 30s, NGO staff, Tokyo workshop).*

If self-testing rapid HIV kits were provided by DVMs located in Japanese saunas, there was a concern that MSM who wanted to have sex after confirming their own infection status may coerce their partner to also use such kits from DVMs. One participant stated *“If someone who is the type of guy I want to have sex with asks me to do a quick test with a kit, I cannot say no. I am really scared of that. If I am young, and an older man asks me to do it, I might not be able to say no” (Gay man in his 20s, NGO staff, Nagoya workshop).*

### Types of HIV testing kits

Many participants stated that postal DBS self-sampling kits, which allow time for careful consideration of whether or not to get tested, would be preferred over rapid self-testing kits where you take your own specimen (blood or oral fluid) and get a result within a short period (approximately 15–20 min), partly due to fears of test coercion by potential sexual partners. That said, some participants felt that MSM with extensive knowledge about HIV infection might prefer a self-testing kit, particularly since results are quickly obtainable (compared to DBS postal kits which are sent off for testing and can take about a week for a result to be returned). Finally, participants expressed that a wider range of test kits available in a DVM (e.g. for other infections, such as syphilis and hepatitis), would be both appealing and beneficial.

### Preferred locations for vending machines

**Figure 1.**
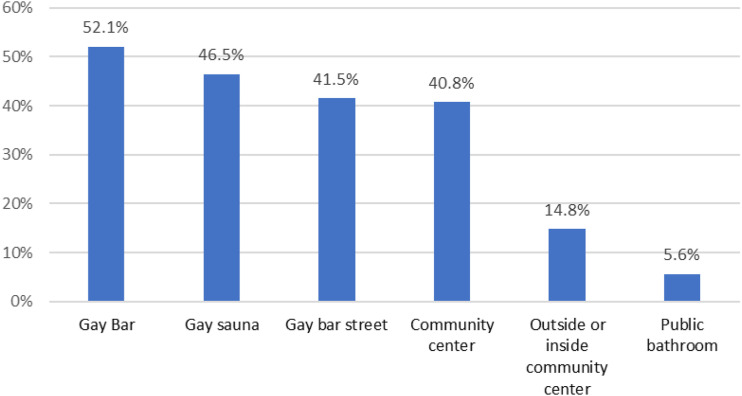
Preferred location for a digital vending machine amongst those who are willing to buy an HIV testing kit from a DVM (*N* = 142; multiple choices were possible).

From many workshop participants, gay bar toilets were mentioned as a desirable location for DVMs. Public toilets, bathhouses, saunas, small club event venues, porn shops, and recreational parks were also listed. The participants suggested the desirability of adapting the colour and surface patterns of the machine to the visual appearance and identity of the locations. “*It is always better to change the colour and design depending on the location, after all, a cute design is very important to attract attention and popularity in the gay community!” (Gay man in his 20s, Volunteer staff, Tokyo workshop).* The quantitative survey also asked about preferred locations from multiple options. Figure 1 shows the preferred DVM locations; for those who wanted to buy testing kits from DVMs, bars were the most preferred location (52.1%), followed by gay saunas (46.5%) and streets outside gay bars (41.5%).

## Discussion

Following the success of the DVM project in the UK, we conducted a mixed-method study to explore the acceptability and feasibility of using DVM-based HIV testing kits amongst MSM in Japan. Overall, our qualitative analysis showed that such a project was highly acceptable amongst the target community. Participants particularly valued the attractive design of the machine and noted that any such design may need to vary, for example depending on the location of the machine to match or blend in with the venue’s milieux. The design team for this feasibility study were also involved in the design of the UK machines.^17,18^ Collaboration between local designers and the design team for adapting the visual appearance of the machine and machine interface was deemed important to increase its acceptability and usage in Japan. The ability of the machine to display comments from social networking sites was also well received. As shown in a UK study,^17^ using DVMs has the potential to increase HIV testing amongst MSM. However, the stigma and discrimination associated with HIV still remains strong in Japan^8^; in fact, in the present study participants expressed strong concerns about being seen whilst purchasing HIV testing kits from DVMs. This reaction gap indicates an important difference between Japan and the UK regarding the stigma associated with HIV infection. This is not only the case in Japan, as similar concerns of “being seen using a machine to get HIV testing kits” were observed in studies amongst ethnic minorities.^24^ Therefore, while using DVMs to promote testing, simultaneous efforts must be made to reduce HIV-related stigma in the longer term.

Fear of receiving a positive test result in isolation and the lack of support and linkage to confirmatory testing in reactive cases were concerns raised in our study that has also been raised in other studies.^17,23,24^ In one Japanese study utilising HIV self-sampling postal tests, support for reactive cases was provided through a unique link accessible only to positive cases. 70% of reactive cases were able to be successfully linked to a confirmatory test and appropriate care.^14^ Therefore it would be important to incorporate similar care pathways to overcome these concerns when using DVMs for accessing testing. Additionally, our findings corroborated that of another study, wherein participants expressed a concern that putting self-testing kits in DVMs may result in test coercion by sexual partners insisting on HIV status confirmation prior to engaging in sexual activities. Therefore, the participants in this study suggested that postal distribution may in some cases be preferable, allowing clients to decide whether they want to take the test in the privacy of their homes. However, there is a possibility of combining both approaches by including self-sampling postal tests within vending machines in addition to self-testing kits. Moreover, these test kits can include tests not only for HIV but also other STIs, including hepatitis and syphilis, as already conducted in the UK.^26^

In the quantitative survey, participants without prior HIV testing experience expressed interest and willingness to try a self-test kit provided by a DVM. Furthermore, our finding that MSM with no testing experience were more likely to want to use the service if it was offered free of charge was consistent with the results of a previous study.^27^Amongst those with previous testing experience, > 60% of the participants wanted to purchase a test from a DVM at a gay sauna, even if it cost 1000 yen. Studies exploring the use of HIV self-testing kits increased the frequency of testing amongst MSM.^28,29^ Fewer people have been tested for HIV in the past year and past 6 months in Japan than in Western countries.^4,5^ Therefore, acquiring DBS postal HIV self-sampling kits (and/or self-testing kits) through vending machines may encourage individuals who have already been tested to test more regularly. As for location preferences, participants in this study preferred having the DVMs in gay commercial and community locations rather than in public locations such as public toilets; this preference differed substantially from that in the UK where participants preferred the latter.^24^

Our findings suggest that the DVMs may help reach communities that require greater access to HIV testing, provided specific concerns are effectively addressed. Presently in Japan, public health centres that are normally responsible for providing free HIV tests are occupied with the prevention and testing of COVID-19; this has led to a 60% reduction in the number of HIV tests performed compared to previous years.^30^ Therefore, it is likely that the demand for contactless HIV testing would remain high. In such circumstances, DVM-based distribution of testing kits might thus prove advantageous.

This study had some limitations which require acknowledgment. First, the quality of data obtained from the workshops and meetings may not be optimal because no method was employed to evaluate or enhance the quality of translation. Second, participants were primarily managers and outreach workers from NGOs with a focus on HIV prevention and sexual health promotion. Thus, they had greater knowledge about HIV and STIs and may not correctly represent the MSM population at risk. Therefore, in the future to assess whether widespread testing by DVMs is acceptable to a wider range of MSM, it would be important to recruit a more diverse MSM population. Indeed, the representativeness of the findings are also limited as a convenience sample was used for the quantitative survey.

In conclusion, the concept of using DVMs to distribute HIV test kits amongst MSM in Japan was found to be both acceptable and feasible and may have the potential to increase access to testing for MSM. Future large-scale studies need to be conducted which could include roll out of digital vending machines (appropriately redesigned for the Japanese context and users) and full-scale evaluation. The impact of this work on HIV testing rates and stigma reduction could be considerable and potentially make an important difference in the lives of MSM in Japan.
